# Exome sequencing reveals independent *SGCD* deletions causing limb girdle muscular dystrophy in Boston terriers

**DOI:** 10.1186/s13395-017-0131-0

**Published:** 2017-07-11

**Authors:** Melissa L. Cox, Jacquelyn M. Evans, Alexander G. Davis, Ling T. Guo, Jennifer R. Levy, Alison N. Starr-Moss, Elina Salmela, Marjo K. Hytönen, Hannes Lohi, Kevin P. Campbell, Leigh Anne Clark, G. Diane Shelton

**Affiliations:** 1 CAG GmbH - Center for Animal Genetics, Paul-Ehrlich-Str. 23, 72076 Tubingen, Germany; 2 Department of Genetics and Biochemistry, Clemson University, 130 McGinty Ct., Clemson, SC 29634 USA; 3 Department of Pathology, University of California San Diego, 9500 Gilman Drive, La Jolla, CA 92093 USA; 4 Howard Hughes Medical Institute, Department of Molecular Physiology and Biophysics, Department of Neurology, The University of Iowa Roy J. and Lucille A. Carver College of Medicine, 4283 Carver Biomedical Research Building, 285 Newton Road, Iowa City, Iowa 52242-1101 USA; 5 Department of Neurology, Roy J. and Lucille A. Carver College of Medicine, The University of Iowa, 4283 Carver Biomedical Research Building, 285 Newton Road, Iowa City, Iowa 52242 USA; 6 Department of Veterinary Biosciences and Research Programs Unit, University of Helsinki, Haartmaninkatu 8, FI-00290 Helsinki, Finland; 7 Folkhälsan Institute of Genetics, Helsinki, Finland

**Keywords:** Muscle, Myopathy, Sarcoglycanopathy, Dog, LGMD

## Abstract

**Background:**

Limb-girdle muscular dystrophies (LGMDs) are a heterogeneous group of inherited autosomal myopathies that preferentially affect voluntary muscles of the shoulders and hips. LGMD has been clinically described in several breeds of dogs, but the responsible mutations are unknown. The clinical presentation in dogs is characterized by marked muscle weakness and atrophy in the shoulder and hips during puppyhood.

**Methods:**

Following clinical evaluation, the identification of the dystrophic histological phenotype on muscle histology, and demonstration of the absence of sarcoglycan-sarcospan complex by immunostaining, whole exome sequencing was performed on five Boston terriers: one affected dog and its three family members and one unrelated affected dog.

**Results:**

Within *sarcoglycan-δ* (*SGCD*), a two base pair deletion segregating with LGMD in the family was discovered, and a deletion encompassing exons 7 and 8 was found in the unrelated dog. Both mutations are predicted to cause an absence of SGCD protein, confirmed by immunohistochemistry. The mutations are private to each family.

**Conclusions:**

Here, we describe the first cases of canine LGMD characterized at the molecular level with the classification of LGMD2F.

**Electronic supplementary material:**

The online version of this article (doi:10.1186/s13395-017-0131-0) contains supplementary material, which is available to authorized users.

## Background

Limb-girdle muscular dystrophies (LGMDs) are a heterogeneous group of Mendelian disorders affecting voluntary muscles of the shoulders and hips [1]. While proximal limb muscles are primarily affected in LGMD, other muscles may degenerate as well, such as the heart and respiratory muscles [1]. Sarcoglycanopathies are a subset of severe, recessive LGMDs (LGMD2C-F) that present in early childhood [2]. There are six known sarcoglycan genes (*SGCA*, *SGCB*, *SGCD*, *SGCG*, *SGCE*, and *SGCZ*); the first four encode single-pass transmembrane glycoproteins (α-, β-, δ-, γ-sarcoglycans) and, along with sarcospan, make up the tetrameric sarcoglycan-sarcospan complex (SGC). As part of the dystrophin-glycoprotein complex, the SGC is critical for maintaining sarcolemmal stability [3]. Mutations in *SGCA*, *SGCB*, *SGCD*, or *SGCG* can result in non-assembly of the SGC and, therefore, the absence of all four sarcoglycans from muscle of affected patients [3, 4]. There are only a handful of low-frequency founder alleles in human populations responsible for sarcoglycanopathies [5]; thus, they are most commonly caused by mutations in compound heterozygosity [6].

In the domestic dog (*Canis familiaris*), selective breeding practices encourage pairing of recessive alleles inherited identical by descent (IBD). Accordingly, dogs have an abundance of recessive disorders [7], including muscular dystrophies [8, 9]. Most canine muscular dystrophies are associated with dystrophin deficiency, and founder alleles have been identified in several breeds [10, 11]. Recently, two independent mutations causing dystrophinopathy were described in Cavalier King Charles spaniels [12, 13].

The first report of LGMD associated with sarcoglycan deficiency in dogs involved three breeds: Chihuahua, Cocker spaniel, and a 7-month-old male Boston terrier from Colorado (case 1), but mutations were not identified [8]. Four years later, sarcoglycanopathy was described again in an unrelated 4-month-old male Boston terrier from Iowa [14] (case 2). All dogs affected with sarcoglycanopathy had a clinical dystrophic phenotype including muscle wasting, gait abnormalities, enlarged tongue, dysphagia, and extremely elevated serum creatine kinase (CK) activities [8, 14]. Pathologic features were consistent with dystrophy, having myofiber degeneration, regeneration, and calcific deposits [8, 14]. Affected dogs lacked muscle α-, β-, and γ-sarcoglycans, confirmed by both western blotting and immunohistochemistry [8, 14]. At the time of evaluation, an antibody reactive with canine δ-sarcoglycan was unavailable.

Here, we describe a sarcoglycanopathy in a third family of Boston terriers from Arkansas in which two puppies (cases 3 and 4) from the same kennel but different litters displayed clinical signs of LGMD, pathological changes consistent with a dystrophic phenotype, and immunohistochemical confirmation of absent or decreased sarcoglycans. To identify the genetic basis for LGMD in the Boston terrier breed, we performed whole exome sequencing (WES) of cases 1 and 3 and related dogs. Evaluation of the sarcoglycan genes revealed, to our surprise, two private deletions in *SGCD*: a 2-bp deletion in exon 6 and a 19.4-kb deletion encompassing exons 7 and 8. Both cause a lack of SGCD, resulting in LGMD2F.

## Methods

### Animals

Clinical details of case 1 were previously published [8]. Biological samples from case 2 were not available. Female Boston terriers, ages 12 and 5 months, and from the same breeder in Arkansas (cases 3 and 4), were evaluated for a chronic history of progressive dysphagia, lack of appetite, drooling, muscle wasting, and greatly enlarged tongues. Both dogs were examined by the same veterinarian in a clinical setting.

DNA was extracted from diagnostic muscle biopsies of cases 1 and 3 and whole blood of unaffected relatives of cases 3 and 4 using the DNeasy extraction kit (Qiagen, Hilden, Germany). Muscle for isolation of DNA was unavailable from case 4. Whole-blood samples or buccal swabs from unrelated, healthy Boston terriers were recruited, and DNA was isolated following the Gentra PureGene protocol (Qiagen, Hilden, Germany) or the MagJet Genomic DNA purification kit (ThermoFisher Scientific, Waltham, USA). Genomic DNAs from unaffected dogs from multiple breeds were available from DNA archives at Clemson University and CAG GmbH.

The dogs in this study were examined and tissues collected in a clinical practice setting with the written consent of their owners. Studies on tissue biopsies and blood samples were approved by the Institutional Animal Care and Use Committees (IACUC) of Clemson University, the University of California San Diego, the University of Iowa, and the Animal Experiment Board in Finland (ESAVI/7482/04.10.07/2015), as well as the Baden-Württemberg veterinary office at the Landratsamt Tübingen Abt. 32: Veterinärwesen und Lebensmittelüberwachung, Tübingen, Germany (Registriernummer: DE 08 416 1038 21).

### Histology and immunofluorescence

Muscle specimens from case 1 were previously obtained as biopsies and archived at −80 °C at the Comparative Neuromuscular Laboratory, University of California San Diego (CNL). Specimens from limb muscles, heart, and tongue were collected by a veterinarian following humane euthanasia at 1 year of age for case 3 and ﻿at 5 months of age for case 4. Muscles were either refrigerated or immersion fixed in buffered formalin and shipped to the CNL. Cryosections from all muscle specimens were processed by a standard panel of histochemical stains and reactions [15].

Antibodies used for immunofluorescence were rabbit antibodies R98 anti-α-sarcoglycan [16], R214 anti-δ-sarcoglycan [17], IIH6 anti-α-dystroglycan [18], R256 anti-sarcospan [19]; mouse antibodies 5B1 anti-β-sarcoglycan [19], 21B5 anti-γ-sarcoglycan [19], AP83 anti-β-dystroglycan [18], anti-dystrophin (AbCam, San Franscisco, CA USA), anti-collagenVI (Fitzgerald Labora﻿tories, Acton, MA USA), and anti-caveolin 3 (BD Transduction Laboratories, San Jose, CA USA); rat anti-perlecan (NeoMarkers, Fre﻿﻿mont, CA USA).

For secondary immunofluorescence, tissues were blocked with 10% goat serum in phosphate-buffered saline, incubated in primary antibody overnight, washed, incubated in Alexa Fluor 488-, 594-, or 647-conjugated anti-rat, anti-rabbit, or anti-mouse antibodies (Life Technologies, San Diego, CA USA ), respectively, and mounted using ProLong Gold mounting media (Life Technologies). For α-dystroglycan, sarcospan, δ-sarcoglycan, and γ-sarcoglycan staining, tissues were fixed in 2% paraformaldehyde, followed by incubations in 100 mM glycine and 0.05% SDS prior to processing as described above. Images were acquired using a VS120-S5-FL slide scanner microscope (Olympus) with VS-ASW software.

### Parentage testing

The Canine Genotypes Panel 1.1 (ThermoFisher Scientific) was used to verify parentage of the experimental dogs. Samples were amplified according to the manufacturer’s instructions and separated and detected on an ABI 3730XL (Applied Biosystems, ThermoFisher Scientific). GeneMarker (Softgenetics, State College, PA, USA) was used to assign peaks and determine genotypes according to ISAG nomenclature.

### Whole exome sequencing

DNA from five Boston terriers (case 1, case 3, and three unaffected relatives of cases 3 and 4) was used for WES performed at CeGaT GmbH (Tübingen, Germany). Genomic DNA (1 μg) from each sample was mechanically sheared to approximately 180–250-bp fragments using a Covaris LE220 Ultrasonicator (Woburn, MA, USA). Fragment sizes were assessed for quality control purposes (Fragment Analyzer, Advanced Analytical Technologics Inc.), and the Agilent SureSelect XT Canine All Exon kit (Santa Clara, CA, USA) supplied the 120-mer biotinylated RNA bases with which the fragment library was hybridized. Magnetic streptavidin beads were used for purification according to the manufacturer’s protocol (Agilent). After amplification of library DNA, adaptors and barcodes for sequencing were added (Illumina), and equimolar amounts of each sample were pooled. Both lanes of a Rapid Flowcell were used to sequence the pool on an Illumina HiSeq2500, generating 2 × 100-bp paired-end sequences, resulting in approximately 6 GB per sample. Illumina bcl2fastq 1.8.2 was used to demultiplex sequencing data, skewer 0.1.116 was used to trim sequencing adapters, and the Burrows-Wheeler Aligner (bwa 0.7.2-r351) was used to map the sequences to the canine genome (CanFam3.1). Samtools 0.1.18 and internal software were used to remove PCR duplicates and low-quality alignments. bcftools (0.1.17) and varscan (2.3.5) and internal software were used to call variants, and a single Variant Call Format (VCF) file was generated for each sample using internal software.

IGV (Integrative Genomics Viewer) [20] and Genome Browse (Golden Helix [21, 22] Inc., USA) were used to visualize data, and the Ensembl dbSNP (Can Fam3.1 version) and whole genome sequences (Clemson) were used to exclude variants.

### Variant characterization and genotyping

#### 2-bp deletion

The 2-bp deletion identified in case 3 was verified by Sanger sequencing, using primers designed to amplify *SGCD* exon 6 (Additional file 1: Table S1). The deletion disrupts a *BcoD1* restriction enzyme site, yielding a 406-bp and a 347-bp product, representing mutant and wild-type alleles, respectively. Unrelated Boston terriers and dogs from other breeds were genotyped using either restriction digest or Sanger sequencing.

#### 19.4-kb deletion

To define the break points of the microdeletion encompassing *SGCD* exons 7 and 8 and the 3′ intergenic sequence, primers were designed in flanking sequences (Additional file 1: Table S1).

For genotyping, primer pairs were designed within the deletion to amplify only wild-type alleles, as well as flanking the deletion for amplification of the mutant allele. Primer pairs were multiplexed for amplification using Phire Hot Start II DNA polymerase (ThermoFisher) and products were resolved by gel electrophoresis. Products were initially verified via Sanger sequencing. The multiplex PCR was used to test unrelated Boston terriers and dogs of other breeds.

## Results

### Clinical findings

**Fig. 1:**
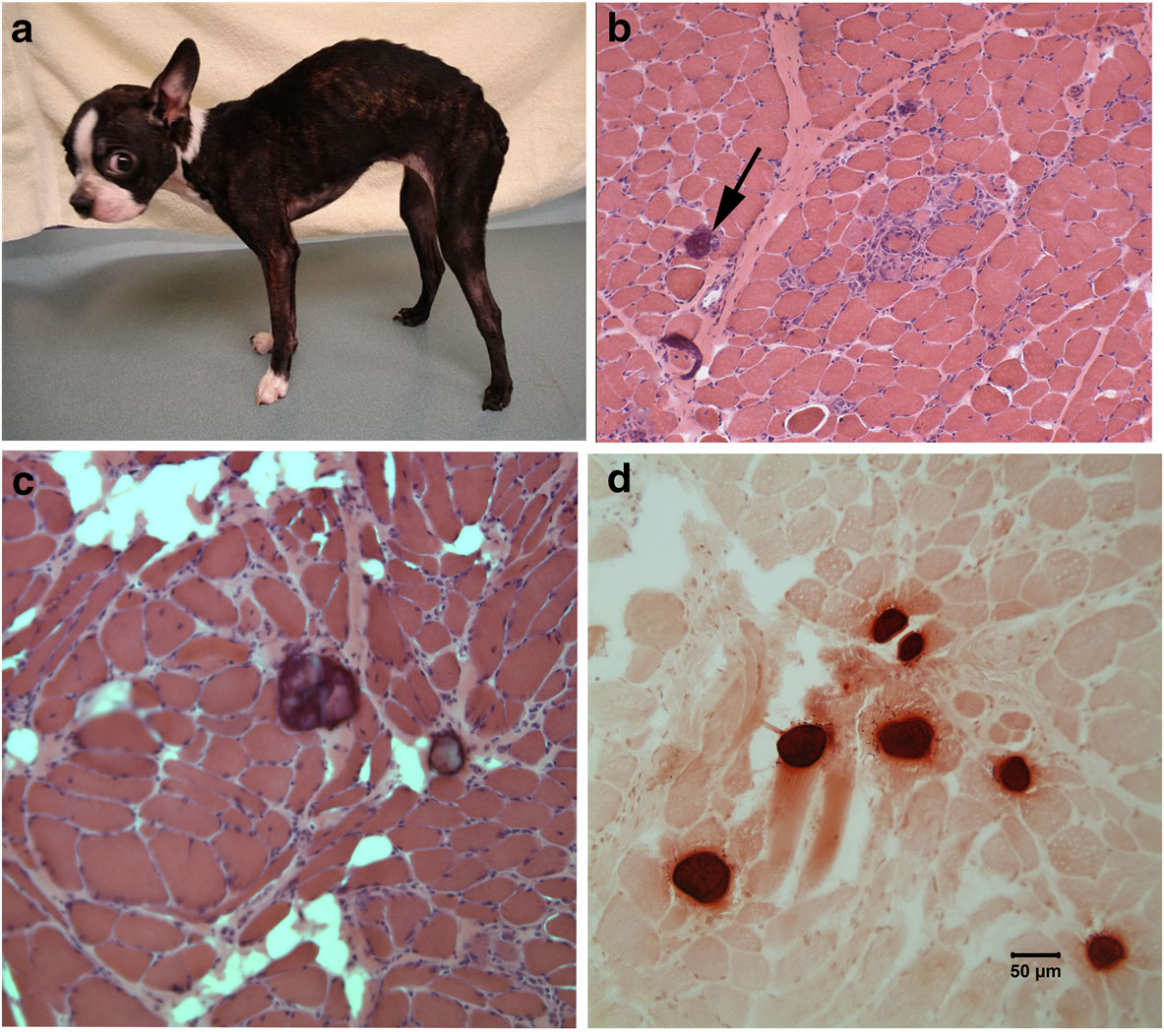
Histopathology of muscle biopsies from a female Boston terrier affected with sarcoglycanopathy (case 3). A hunch back stance was evident in the dog (**a**). H&E stained cryosections from a representative limb muscle (**b**) showed degenerative changes and calcific deposits (*black arrow*). Similar degenerative changes and calcific deposits were observed in the tongue (**c**). The calcific deposits in the tongue were highlighted *bright orange* using the alizarin stain for calcium (**d**)

Muscle wasting, dysphagia, exercise intolerance, lethargy, and failure to thrive were accompanied by progressive gait abnormalities including a short, stilted gait in cases 3 (Fig. 1a) and 4. While there was no clinical indication of cardiomyopathy, specific evaluations for heart disease by a veterinary cardiologist were not performed. Clinical chemistry included markedly elevated activities of serum alanine aminotransferase (ALT; 900 IU/L, reference range 10–110 IU/L), aspartate aminotransferase (AST; 920 IU/L, reference range 16–50 IU/L), and creatine kinase (CK; >10,000 IU/L, reference range 50–275 IU/L). Progression of clinical signs necessitated euthanasia at approximately 1 year of age for case 3 and 5 months of age for case 4.

### Histology and immunofluorescence

**Fig. 2:**
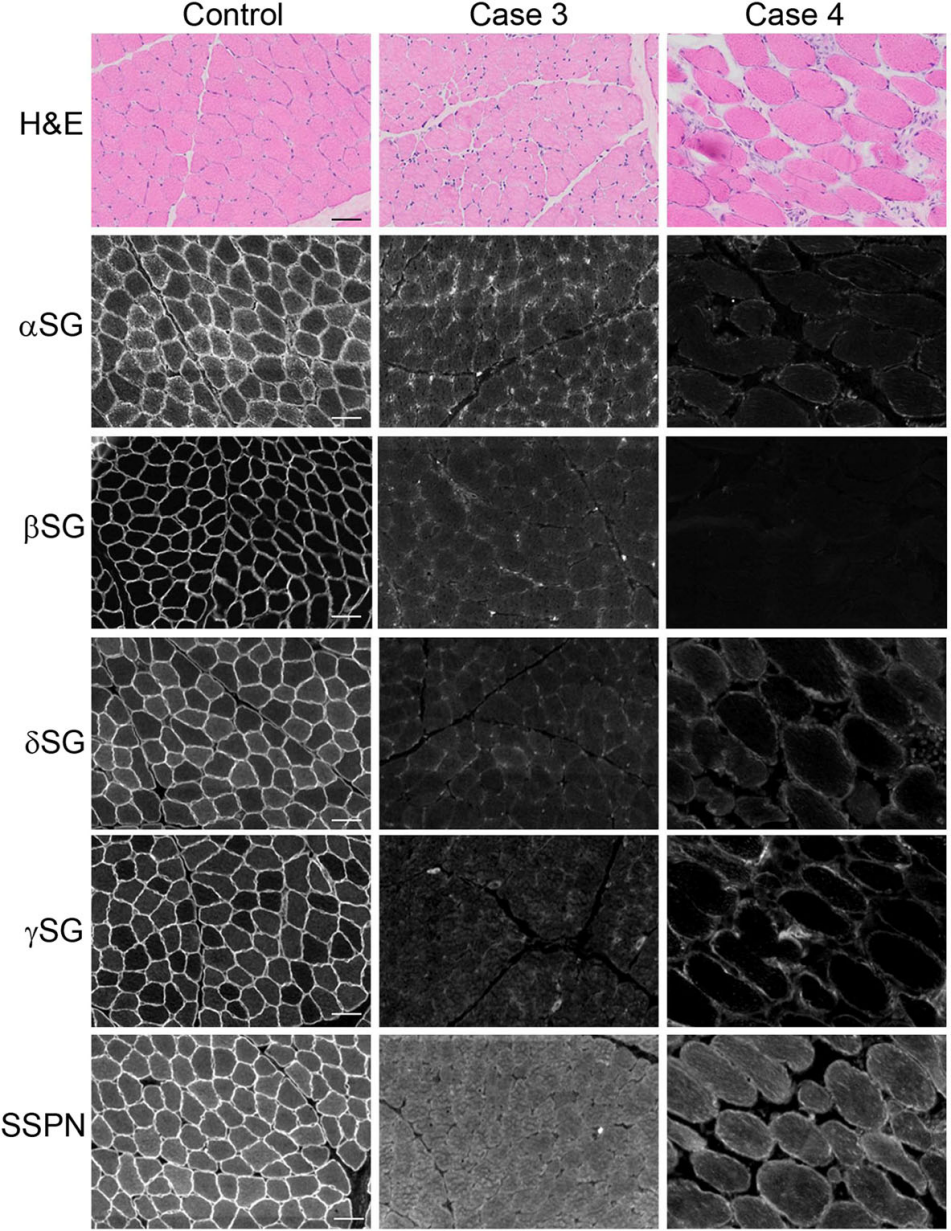
Loss of SGC staining in cases 3 and 4. Representative H&E and immunofluorescence of cryosections from the muscle of cases 3 and 4, as well as of a control dog muscle. In the control muscle, antibodies to the SGC (α-, β-, δ-, γ-sarcoglycans: αSG, βSG, δSG, γSG), as well as sarcospan (SSPN), localize to the sarcolemma of the muscle fibers. Staining from each of these antibodies is reduced in muscle from cases 3 and 4

**Fig. 3:**
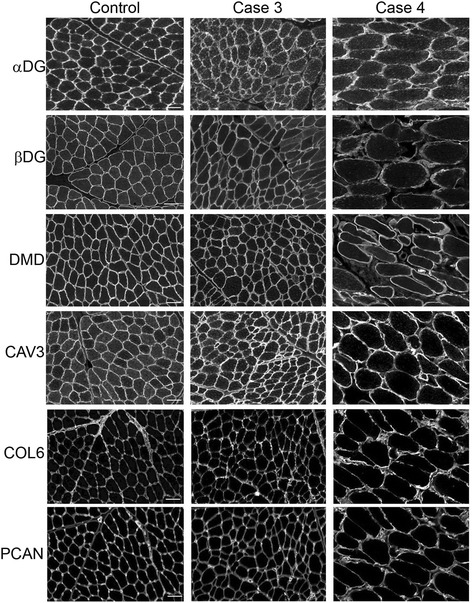
Representative immunofluorescence of cryosections from muscle of cases 3 and 4 and control dog muscle. Staining of α-dystroglycan (αDG), β-dystroglycan (βDG), dystrophin (DMD), caveolin 3 (CAV3), collagen VI (COL6), and perlecan (PCAN) in cases 3 and 4. Antibodies to α- and β-dystroglycans, dystrophin, caveolin 3, and perlecan demonstrate sarcolemmal localization and intensity that is comparable to control tissue. An antibody to collagen VI shows increased localization to the endomysium compared to the control tissue, consistent with endomysial fibrosis

A dystrophic phenotype including degeneration, regeneration, and calcific deposits was evident in the skeletal muscle (Figs. 1b) and tongue (Fig. 1c, d). Heart muscle was histologically normal (left ventricle, not shown) from cases 3 and 4. Immunofluorescence staining of muscle cryosections showed markedly reduced or absent localization of α-, β-, γ-, and δ-sarcoglycans and sarcospan in cases 3 and 4 (Fig. 2). In contrast, staining for localization of α- and β-dystroglycans, dystrophin, caveolin 3, and perlecan was similar to control muscle (Fig. 3). Staining for collagen VI was increased in the endomysium compared to the control tissue, consistent with endomysial fibrosis. Results of histology, immunofluorescence staining, and western blotting of case 1 were described previously [8]. Staining for localization of δ-sarcoglycan in case 1 was performed on archived muscle cryosections and was similarly absent (not shown).

### Parentage testing

Parentage testing was performed to determine relationships between case 3 and three other dogs obtained from the same breeder. One relative was confirmed to be the dam of case 3 and is referred to hereafter as the obligate carrier. The test excluded the remaining two dogs from being full siblings of case 3 or progeny of the obligate carrier. Their relationship to the other dogs or to one another could not be determined.

### Variant identification from WES

Disruption of any one of the sarcoglycans results in reduced immunostaining of the entire SGC, both in LGMD patients and in animal models of sarcoglycanopathy [23–25]. Therefore, genetic sequencing was necessary to identify the defective sarcoglycan gene.

#### 2-bp deletion

The candidate genes (*SGCA*, *SGCB*, *SGCD*, *SGCG*) were sequenced to an approximate depth of 30X. For each gene, we manually screened the VCF file in IGV for variants fitting a pattern of inheritance consistent with a rare recessive allele. We expected both affected dogs to have inherited the causal mutation IBD from a common ancestor; therefore, we searched for variants homozygous in cases 1 and 3, heterozygous in the obligate carrier, and heterozygous or homozygous wild-type in the two relatives. No variants fit these criteria.

**Fig. 4:**
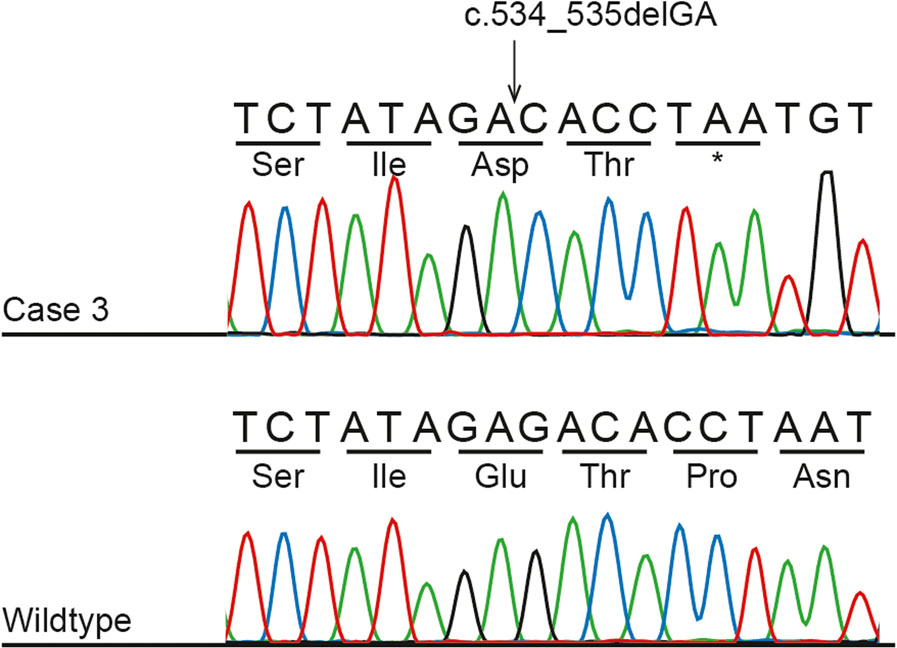
Electropherogram showing the 2-bp *SGCD* deletion in case 3. The *top panel* shows the sequence from case 3, while the *lower panel* shows the sequence from a healthy non-related Boston terrier. The *SGCD* c.534_535delGA mutation leads to a frameshift and a premature stop codon two amino acids later

Because there was no known relationship between cases 1 and 3, we considered that they may have different genetic causes for LGMD. Thus, we excluded case 1 and searched again for the same pattern. Only one variant fit the pattern: a 2-bp deletion in exon 6 of *SGCD* (Fig. 4). We validated the deletion through Sanger sequencing and determined that, in addition to the obligate carrier, one relative was heterozygous. The deletion predicts the substitution of an aspartate for a glutamate (E178D) and creates a frameshift, leading to a premature stop codon two amino acids later (P180X) (Fig. 4). We genotyped 199 Boston terriers and 127 dogs from 33 other breeds; none possessed the deletion.

#### 19.4-kb deletion

Using BAM files, we reexamined each candidate gene for variants homozygous in case 1 and absent from the other Boston terriers. This approach revealed a complete absence of reads from the final two exons of *SGCD* (7 and 8) in case 1, which was not apparent from the VCF file. No other variants fit the pattern. We hypothesized that the absence of reads represented a microdeletion and designed three primer pairs flanking exons 6, 7, and 8. PCR amplification yielded a product for exon 6 but not for exon 7 or 8 in case 1, providing further support for the presence of a deletion.

**Fig. 5:**
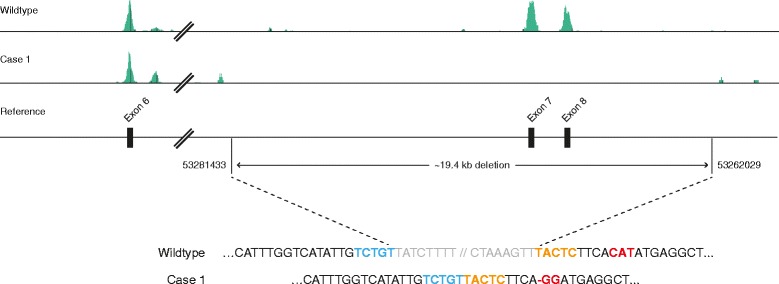
Schematic and sequence showing the breakpoints of the 19,403-bp *SGCD* deletion in case 1. Note that *SGCD* is annotated on the minus strand. Whole exome sequence from a healthy dog and case 1 are aligned to the reference genome, visualized in Golden Helix GenomeBrowse ® [21, 22]. Case 1 has no coverage of exons 7 and 8 and flanking regions. Sequence of the wild-type and case 1 alleles show the precise breakpoints. Nucleotides 5′ and 3′ of the breakpoint are in *bold blue* and *orange* typeface, respectively. A substitution (chr4:53262020-53262018, CAT > GG) is found 9 bp downstream of the microdeletion and is shown in *bold red* typeface

It was not possible to characterize the deletion directly from WES because intergenic and intronic sequences are minimized. Sequence coverage indicated that the deletion was between *SGCD* exon 6 and *TMD4*. Furthermore, sporadic intronic and intergenic fragments were present 5′ of exon 7, beginning at chr4:53282570, and in the 3′ UTR, beginning at chr4:53261359, suggesting a maximum deletion size of 21,211 bp. Primer pairs flanking this estimated deletion size yielded large products (~3–5 kb), indicating a deletion approximately 2 kb smaller than suggested by WES. Sanger sequencing of the breakpoint revealed a substitution (chr4:53262018-53262020, ATG > CC), followed by 9 bp that were unchanged before a deletion of 19,403 bp (chr4:53262030-53281432) (Fig. 5). We genotyped 201 Boston terriers and 91 dogs of 19 other breeds and did not find any carriers.

## Discussion

Sarcoglycanopathies in humans are rare genetic disorders, with an incidence of one in every 178,000 human births [26]. To date, only small animal models are available for study: gene-targeted mouse models for α-, β-, δ-, and γ-sarcoglycanopathy [27] and a spontaneous hamster model for δ-sarcoglycanopathy [28, 29]. Here, we have demonstrated that a naturally occurring muscular dystrophy in a Boston terrier family is a sarcoglycanopathy, consistent with two previously published case reports in the breed. Given that cases have been described in three Boston terrier families, we expected a single recessive allele, present at a very low frequency within the breed, to underlie all cases. Instead, we uncovered independent mutations in the two families studied herein. Unfortunately DNA from case 2 [14] was not available to determine whether this dog shared one of the mutations described herein, a different mutation in *SGCD*, or a pathogenic variant in another gene.

Both families possessed mutations in *SGCD*, which encodes δ-sarcoglycan. Canine *SGCD* is located on CFA 4 and organized into eight exons that form a 1297 bp mRNA transcript [30]. Human (XP_016865213.1) and dog (XP_013968526.1) amino acid sequences share 98% identity. Despite being the largest of the sarcoglycan genes, *SGCD* least commonly causes sarcoglycanopathy, with the majority of human cases attributed to changes in *SGCA* [31]. Thus, it is not only surprising that the Boston terriers had independent mutations causing sarcoglycanopathy, but that both had pathogenic alleles of *SGCD*. Curiously, the only other naturally occurring model of a sarcoglycanopathy, the Syrian hamster, also harbors an *SGCD* deletion [29].

Mutations of *SGCD* cause LGMD2F, and although clinical presentation is largely similar among the four sarcoglycanopathies, this is the only subtype not consistently characterized by concomitant cardiomyopathy [1]. The absence of heart involvement in Boston terriers is consistent with this classification; however, because the affected dogs were euthanized at an early age it is unknown if muscle degeneration would have progressed to involve the heart.

Immunohistochemistry illustrated a lack of the SGC in both cases, but provides no indication as to whether SGCD is abnormal or absent altogether. Due to limited sample availability, collected tissues were prioritized for histopathological analysis and genomic DNA sequencing. RNAs were thus unavailable to investigate the consequence of the deletions on *SGCD* transcripts. The 2-bp pair deletion predicts a premature stop codon in exon 6, possibly causing nonsense-mediated decay. The 19.4-kb microdeletion eliminates the last two exons of *SGCD*; the complete loss of an SGCD exon is rare [5]. It is hypothesized that exon 6 would splice to one or more cryptic sites, triggering either nonsense-mediated decay and/or the production of mutant protein. It is likely that mutant SGCD would cause assembly of the SGC to fail, resulting in LGMD [3, 32].

WES is a cost-effective method for the sequencing of multiple family members and has been used successfully to identify LGMD mutations in humans [33]. It was an advantageous choice over transcriptome sequencing in this study because *SGCD* transcripts would have been absent in case 3 and possibly case 1 as well, necessitating additional sequencing of *SGCD* to identify the causative mutations. In dogs, WES has led to the identification of alleles underlying progressive retinal atrophy, primary angle closure glaucoma, and nemaline rod myopathy using small numbers of related cases [34–38] but is not ideal for detecting intergenic deletions or genomic rearrangements [39]. The development of improved WES enrichment kits for dogs [39, 40] will facilitate future detection of disease variants in canine models.

## Conclusion

The identification of canine models of disease holds promise for new advances in the understanding and treatment of analogous human diseases. For example, the well-characterized Golden retriever model of Duchenne muscular dystrophy (DMD) has proven to be an invaluable resource for gene therapy and other trials [41, 42]. Here, we have clinically and genetically characterized the first large animal model of sarcoglycanopathy.

## Additional file

Additional file 1: Table S1. Primers used to define and genotype SGCD mutations. (DOCX 12 kb)

## Abbreviations

bp: Base pair
CAG: Center for Animal Genetics, CAG GmbH
CNL: Comparative Neuromuscular Laboratory, University of California San Diego
DMD: Duchenne muscular dystrophy
DNA: Deoxyribonucleic acid
H&E: Hmatoxylin and eosin
IBD: Identical by descent
IGV: Integrative Genomics Viewer
LGMD: Limb-girdle muscular dystrophy
PCR: Polymerase chain reaction
SGC: Sarcoglycan complex
SGCA: α-sarcoglycan
SGCB: β-sarcoglycan
SGCD: δ-sarcoglycan
SGCE: ε-sarcoglycan
SGCG: γ-sarcoglycan
SGCZ: ζ-sarcoglycan
UTR: Untranslated region
VCF: Variant call format
WES: Whole exome sequencing
