# Supplementary material for: Exome sequencing reveals independent SGCD deletions causing limb girdle muscular dystrophy in Boston terriers
Source: Skelet Muscle. 2017 Jul 11;7:15. doi: 10.1186/s13395-017-0131-0 (PMC5506588; doi:10.1186/s13395-017-0131-0)
Supplement: Additional file 1: Table S1. — Primers used to define and genotype SGCD mutations. (DOCX 12 kb) [file 13395_2017_131_MOESM1_ESM.docx]

| Primer Set | F primer sequence | R primer sequence | Expected size (bp) | Purpose |
| --- | --- | --- | --- | --- |
| Exon 6 | CGCTGGCAAAATCCGTGCTTTGGG | GGCAATCTCCTCCTCCAGACCCCC | 420 | AR mutation genotyping |
| SGCD CO mut | GTGTCGAGGGTGAAATAAGTGA | ATTAGGGAGGCCAGTCTATTCA | 678 | CO mutation genotyping |
| SGCD CO wt | CACATTATGGAGTCTGAGCTAACT | Ccatcactaaatagtctcagctgt | 434 | CO mutation genoptying |
| CO breakpoint | GGCTTCATAGATAGTGGGCTG | ATTTCCATCTCAAACTCATTCAA | Wt = 20681 (no amplification)  CO mutant: 1275 | CO breakpoint determination |
| Exon 7 | ACCTTCTGATTGGCTCCTCT | TTACTTCCTCCCTCATAAACTTG |  |  |
| Exon 8 | CCACCCACACACATACACACA | TGCTCACTTAGGCTGAACACA |  |  |
